# The Implementation of Infection Prevention and Control Procedures in Primary Care During the COVID-19 Pandemic: A Qualitative Study of Nursing Roles

**DOI:** 10.1155/jonm/6634676

**Published:** 2025-05-18

**Authors:** Samina Idrees, Maria Mathews, Lindsay Hedden, Julia Lukewich, Emily Gard Marshall, Kelly Kean, Rhiannon Lyons, Jamie Wickett, Leslie Meredith, Dana Ryan, Sarah Spencer, Émilie Dufour, Paul Gill

**Affiliations:** ^1^Department of Family Medicine, Schulich School of Medicine & Dentistry, Western University, London, Ontario, Canada; ^2^Faculty of Health Sciences, Simon Fraser University, Burnaby, British Columbia, Canada; ^3^Faculty of Nursing, Memorial University, St. John's, Newfoundland and Labrador, Canada; ^4^Department of Family Medicine Primary Care Research Unit, Dalhousie University, Halifax, Nova Scotia, Canada; ^5^College of Registered Nurses of Newfoundland and Labrador, St. John's, Newfoundland and Labrador, Canada

**Keywords:** COVID-19, infection prevention and control, nurse roles, nursing, pandemic response, primary care, qualitative research

## Abstract

**Introduction:** During the COVID-19 pandemic, primary care practices felt poorly supported by existing infection prevention and control (IPAC) guidelines, which focused primarily on acute care facilities. This issue was further complicated by insufficient provision of personal protective equipment in primary care settings, which limited clinic capacity and the ability of primary care to provide in-person services. Nurses play an integral role in the implementation of IPAC procedures and the provision of ongoing primary care during a health crisis; however there is limited literature related to nurses' roles in the enactment of IPAC procedures in primary care settings. This paper aims to describe primary care nurses' experiences and roles in implementing IPAC during the COVID-19 pandemic.

**Design:** Qualitative analysis of interviews as part of a larger mixed methods case study.

**Methods:** We conducted semistructured qualitative interviews with primary care nurses across four Canadian regions in the provinces of British Columbia, Ontario, Nova Scotia, and Newfoundland and Labrador. During the interviews, we asked participants to describe the roles they enacted during the various stages of the pandemic, any facilitators and challenges they encountered, and the potential roles that nurses could have played. We employed a thematic analysis approach, and, for the purposes of this paper, we analyzed themes relevant to the implementation of IPAC.

**Results:** We interviewed 76 nurses across the four regions and identified two overarching themes: (1) nurse-led transformation of clinic operations and (2) impact on workload. Primary care nurses developed and implemented IPAC policies, educated staff, and made critical decisions about patient care, often out of necessity and ahead of regional guidelines. In addition, nurses adapted workflows, managed supplies, and balanced in-person and virtual care to protect both patients and staff from COVID-19 exposure.

**Conclusion:** Despite the additional responsibilities and challenges that nurses faced in response to evolving guidelines, their IPAC efforts were pivotal in maintaining primary care clinic operations during the pandemic. The findings from this study underscore primary care nurses' capacity to adapt and apply evidence-based practices and demonstrate the need for better pandemic planning to support primary care. IPAC guidance documents, suitable for primary care settings and informed by experiences from the COVID-19 pandemic, should be included in future pandemic plans.

**Implications for Nursing Management:** Our findings highlight the need for stronger institutional support and preparedness for primary care nurses during a pandemic. Nursing management should ensure that IPAC responsibilities are explicitly recognized within primary care nursing roles and supported through ongoing training, resource allocation, and standardized protocols. Proactively integrating IPAC into primary care practice and strengthening these supports will enhance future health crisis preparedness while mitigating nurse burnout and promoting sustainable workforce capacity.

**Summary**

• **Clinical Relevance:** Primary care nurses played an instrumental role in developing and enforcing IPAC protocols and enhancing clinical safety during the pandemic, underscoring the need to leverage nursing expertise in managing infectious disease outbreaks.
• There is a need for structured support and resources for primary care nurses to enhance pandemic preparedness and response, ensuring that clinics can swiftly and effectively adapt to future public health crises.

## 1. Introduction

Infection prevention and control (IPAC) refers to a set of processes, procedures, and policies that aim to minimize the spread of communicable diseases in a number of settings, including healthcare [1]. IPAC includes, but is not limited to, various procedures such as screening, testing, disinfecting, the use of personal protective equipment (PPE), physical distancing, and virtual care use. During the COVID-19 pandemic, primary care practices were poorly supported by existing IPAC guidelines which focused on acute care facilities and therefore had to be rapidly adapted by providers, including family physicians and nurses, for application in primary care settings [2–7]. For example, while guidelines recommended limiting in-person appointments in an effort to minimize the spread of COVID-19, this created a challenge for primary care practices as they were forced to balance conflicting priorities in order to ensure routine patient care remained accessible [6]. The lack of PPE provision for primary care settings further complicated IPAC implementation and limited the capacity of primary care clinics to provide in-person care [2, 3, 6, 8–13]. Over time, and as more information regarding transmission of the novel virus became available, regional health authorities in Canada provided guidance on how to mitigate virus transmission across different healthcare settings, including IPAC guidelines that were better tailored to clinic-based primary care practices [14–18].

In Canada, nurses play a critical role in primary care provision and their broad scope of practice and experience uniquely positions them to manage infection risks effectively [19]. There are three nursing designations employed within primary care across Canada [20]. Nurse practitioners (NPs) have a master's degree in nursing and can work independently or in collaboration with other healthcare providers to diagnose and treat various medical conditions. Registered nurses (RNs) generally have a baccalaureate degree in nursing (e.g., BScN) and can conduct patient assessments, collaborate with other providers to develop care plans, administer medications, collect samples for testing, and monitor the health of patients within their scope of practice. Licensed practical nurses (LPNs), also known as registered practical nurses (RPNs) in Ontario, have a college diploma in practical nursing and are autonomous practitioners working collaboratively with other care providers and are authorized to make care decisions independently with clients who have established plans of care and where outcomes are predictable [21].

In contrast to single-provider practices, interprofessional primary care teams were better able to adapt their practices andtheir provision of care during the pandemic [7, 22, 23], including meeting new and evolving IPAC requirements. Although nurses are identified as IPAC providers [24], there is limited understanding in the literature about the roles they play in implementing IPAC in primary care settings. To address this gap, this paper aims to describe nurses' experiences and roles in implementing IPAC within primary care settings during the COVID-19 pandemic.

## 2. Design

This qualitative descriptive analysis stems from a larger, multimethods project examining the roles and experiences of primary care nurses during the COVID-19 pandemic. Qualitative studies are well-suited for exploring participants' perceptions and experiences, especially when relatively little is known about a topic such as primary care nurse roles in the COVID-19 pandemic response. We used qualitative description which allowed us to remain close to the descriptions and language of participants, avoiding overly abstract or conceptual interpretations [25, 26].

## 3. Materials and Methods

We conducted semistructured qualitative interviews with primary care nurses in health regions across four Canadian provinces: the Interior, Island, and Vancouver Coastal health regions in British Columbia, the Ontario Health West region in Ontario, the province of Nova Scotia, and the province of Newfoundland and Labrador. This paper focuses on interview data specific to the role of primary care nurses in developing and implementing IPAC guidelines.

### 3.1. Recruitment

We included nurses (NPs, RNs, and LPNs/RPNs) who practiced in a primary care setting during the COVID-19 pandemic between March 2020 and January 2023. We excluded nurses who worked in primarily nonclinical roles (i.e., academic, research, or administrative), and nurses who did not have active practice licenses.

We used maximum variation sampling to recruit nurses across a diverse range of characteristics, including gender, years in practice, practice type, payment model, and community size [27]. We identified potential participants using online sources, such as available practice lists and search portals managed by professional or regulatory colleges. We also worked with professional colleges to promote our study to their members using social media, organizational newsletters, and email.

We identified clinics employing primary care nurses through website searches. We used the email listed on the clinic website to relay study information and invitations to participate. We used snowball sampling in regions where it was permitted by the institutional ethics board. We continued recruitment until we reached the point of data saturation [27, 28]. This was determined through consultation with the research team and defined as the point at which further interviews no longer provided additional insights.

### 3.2. Data Collection

Researchers and research assistants in each region with graduate-level preparation and prior interviewing experience conducted the interviews. In each interview, we asked participants about the roles they took on during different stages of the pandemic [29], the facilitators and barriers they encountered, and additional roles they felt they could have filled during this time. The interview guide is presented in Supporting Information. We also collected relevant demographic information, including participant gender, years in practice, payment model, and practice characteristics. We conducted the interviews in a semistructured fashion and made modifications to the interview guide for each region to reflect differences in health systems, nursing roles and responsibilities, and pandemic impact and response. The interviews took place over the phone or through videoconference (Zoom Video Communications Inc.), depending on participant preference. The interviews were audio-recorded and transcribed verbatim. We redacted identifiable information from interview transcripts and included interviewer field notes to aid in data analysis.

### 3.3. Data Analysis

We analyzed the data thematically [30], which aligns with the naturalistic approach of qualitative description [31]. The initial coding of the data proceeded with research personnel in each region reviewing a selection of four transcripts (one from each province and at least one from each nursing designation) to develop a coding template that reflected key ideas and messages in the data. The team members from all four regions then met multiple times over a series of weeks to compare coding and refine coding labels and definitions. We resolved disagreements through discussion and consensus, until we arrived at a unified coding template with consistent terms and descriptions. The unified coding template was used to code interview transcripts across each of the four provinces. Research assistants used the unified coding template to code interview transcripts across each of the four provinces. We used NVivo (QSR International), qualitative research management software, to code the data and produce node reports. The co-authors reviewed the node reports related to the implementation of IPAC guidelines and other identified themes (i.e., recurring ideas and concepts) in the data and sorted the quotations along these themes. Through meetings and reviews of manuscript drafts, the co-authors discussed the meanings of the individual themes and their relationship to each other. For example, we noted that both changes to nurses' workflow and the evolving nature of public health guidelines affected nurses' workload. We compared and contrasted across interviews to identify similarities and differences in findings across nursing professions, provinces, and genders.

### 3.4. Rigor

We took several steps to promote methodological rigor and the trustworthiness of the findings [27, 28, 30, 32]. To enhance the credibility of the findings, we used trained interviewers and pretested interview questions. Prior to each interview, we provided a chronology of the pandemic to participants and referred to the chronology during the interview to enhance participant recall. We confirmed our understanding of participants' perspectives during interviews to ensure accurate interpretation. We also describe nonconfirming examples in our results. To enhance the repeatability of the study, we maintained an audit trail of recordings, transcripts, field notes, drafts of the coding template, resolutions of coding disagreements, node reports, and manuscript drafts. We have also provided the interview guide as Supporting Information to support future replication of our methodology. We promoted transferability by providing background on the regions in the study, the qualifications of the three nursing designations, and the characteristics of participants in the study [32]. We also use rich descriptions by providing the context of quotes and triangulating findings with other research on primary care providers' responses during the COVID-19 pandemic in Canada [6, 29] and the lived experiences of primary care providers on our research team. To enhance confirmability, in addition to the audit trail described above, multiple individuals developed the coding template and coded data. Moreover, we took steps to ensure consistency of coding by comparing the coding of a subset of transcripts and clarifying code descriptions. Lastly, we encouraged reflexivity among team members.

### 3.5. Positionality

The study was conducted by an interdisciplinary team of researchers and healthcare professionals, including experts in primary care, public health, nursing, health services research, and epidemiology. We drew upon the diverse skillset of our study team to develop study questions and contextualize study findings.

### 3.6. Ethics

This study was approved by the Research Ethics Boards at Research Ethics British Columbia, the Health Research Ethics Authority of Newfoundland and Labrador, the Nova Scotia Health Authority Research Ethics Board, and Western University's Research Ethics Board.

## 4. Results

We interviewed a total of 76 nurses across the four provinces (Table 1). The majority of participants identified as women (*n = *72). The sample included 24 NPs, 37 RNs, and 15 LPNs/RPNs. The different nursing designations were represented to varying degrees across the four regions. The participants provided care in various settings, including rural and urban communities, and had an average of 14.9 years in practice. We identified two overarching themes related to the implementation of IPAC by primary care nurses: (1) nurse-led transformation of clinic operations and (2) impact on workload (Table 2). We compared participants' roles and experiences implementing IPAC across the four provinces and three nursing designations and found that themes were consistently expressed by all participants, with no notable differences between groups.

### 4.1. Nurse-Led Transformation of Clinic Operations

Nurses were integral to the transformation of clinic operations and procedures to align with the emerging IPAC guidelines. They played an active role in (1) the development and implementation of IPAC policies and protocols, (2) teaching and education of team members, and (3) virtual care decision-making.

#### 4.1.1. Policy and Protocol Development

Participants described key roles they played in developing IPAC policies and protocols for their clinic (e.g., developing screening questions and cleaning protocols): *“… my work with the (team of providers within the clinic that focused on pandemic-related issues) … was kind of policy development within the clinic… (developing) screening and PPE (protocols) and we changed our entire workflow…”* (ON17 NP). Over the course of the pandemic, regional health authorities released guidance to aid clinics in developing appropriate IPAC policies and procedures. The application of this guidance varied by clinic, reflecting the importance of nurses' decision-making roles: *“*… *(the regional health authority) every day would send out almost a new COVID-19 policy in email and we would always follow that protocol of course… but specific policies for the clinic, completely up to us and our manager”* (NL07 LPN). Nurses adapted clinic operations to align with emerging IPAC guidelines, while at the same time considering clinic capacity, resources, and infrastructure. For example, one nurse described adapting normal clinic operations to allow for potentially infectious patients to be seen in-person, while still adhering to public health recommendations regarding isolation and distancing:

“*I was tasked with the responsibility of creating a workflow or a process for dealing with COVID-19 patients…. If somebody's got (any) respiratory (symptoms), they phone us when they get here, they come around to the back door, I take them into the closest office exam room, which is just right by the back door, which is set up so that it's our respiratory office. And we've got our full PPE…”* (BC07 RN)

Responsibilities for developing IPAC guidelines and procedures for the clinic were formally assigned, or informally assumed, by nurses who identified a need to protect staff and patients from COVID-19 exposure. In Nova Scotia, a NP observed a need for more comprehensive cleaning of high-touch surfaces and directed their clinic to hire a cleaner: *“…there was a lot of logistic kind of weird stuff that I got involved in… making (the clinic get) a cleaner so that they would clean up some of the stuff. People were coughing and barking all over the doors…”* (NS05 NP). Likewise, a RN from British Columbia described taking a proactive approach to mitigate disease spread among staff at the clinic by discouraging reusing masks that had been placed on potentially contaminated surfaces:

*“I put different things on the boards and highlighted things like “you shouldn't be putting your mask down on the kitchen table and then putting it back on. You need a new one, I don't care if they told you to hold on to your mask all day, three months ago. That's not what is best practice'””* (BC05 RN).

The IPAC guidance from regional health authorities was sometimes described as lagging behind measures that nurses had already taken to limit disease spread in their clinics:

*“We were trying to see all the sick people, (and we wanted to) separate (them) from the well-people… so we would have a clean provider and a dirty provider… (and) we would alternate. I would say that one of the hardest things was that we were having to come up with our own solutions… Two weeks after we've been doing whatever we're going to do, the Ministry would say, “Hey, you should do this,” we're like, “Yeah, thanks. Done. And we've already changed on to the next one and you guys aren't here yet””* (ON20 NP).

#### 4.1.2. Teaching and Education

Nurses' IPAC roles also incorporated important staff teaching, quality assurance, and supply management components. The effective implementation of IPAC was contingent upon clinic staff member's awareness of and ability to enact IPAC protocols and procedures. Participants often described being responsible for ensuring staff was appropriately trained and adhering to protocols: *“… (As the team lead I) would help out with the staff that (were) there, any nurses that were swabbing, if they had any questions about how the process worked… (and making) sure people were donning and doffing the PPE properly”* (NL11 LPN). These responsibilities could extend to nurses' roles in community-based healthcare settings such as COVID-19 hotels or long-term care homes:

*“I witnessed the nursing and the health professionals in the long-term care (home) walking around with gloves on their hands. So, I had to teach them, “stop, don't touch your face… and if you touch your face, you gotta wash your hands””* (ON01 RN).

Although some nurses mentored colleagues through observation and helping to answer questions, others took a more targeted approach, by using informative posters to highlight appropriate techniques and placing PPE carts in low-risk areas:

*“I set up a (PPE) cart… in rooms that were off to the side so they had less traffic… I printed up posters to show you how to gown, how to glove, mask… how to get rid of your PPE without contaminating yourself”* (NS05 NP).

Nurses' teaching of staff was not limited to clinical members of the team, as several participants described the importance of front-desk staff in implementing IPAC, and the corresponding education role nurses played in training them to do so: *“I taught the clerical (staff) everything (they had) to do, with the waiting rooms and screening patients and how to schedule them in the system…”* (NL06 RN).

#### 4.1.3. Triaging Virtual and In-Person Care

In some primary care clinics, nurses also helped determine which patients should receive virtual consultations as opposed to in-person visits. These decisions required nurses to take into consideration a variety of different factors, including the patient's symptoms, screening results, risk profiles, available resources (e.g., PPE), and clinic capacity: *“We'd have prescreened our patients not only for symptoms of COVID-19 but also for whether or not their concern warranted an in-person exam”* (ON07 NP). This role was an important part of IPAC, and effective decision-making within this context provided an added layer of protection for staff and patients by limiting contact with potentially infected individuals. Nurses described taking a proactive approach to protecting at-risk patients. In Nova Scotia, a NP described leading discussions on how to prevent vulnerable patients from being infected while in the clinic: *“… I hold (clinical meetings) twice a month… (and we were) trying to decide how to limit access, not limit access, but sort of try to keep our chronically ill patients that may be immunocompromised from coming in contact with persons that may be infectious”* (NS05 NP). In Ontario, NPs had similar decision-making roles, weighing the benefits and risks associated with scheduling in-person visits:

*“… if you were a transport truck driver and you traveled over the border… we were doing those (appointments) by telephone just because of the risk… that was just one example… when those situations came up, I helped to assist people in decision-making with that and how we should proceed… kind of using it as a tool to look back on to guide us”* (ON26 NP).

There was variability in how closely a nurse followed public health guidelines relating to the provision of in-person versus virtual care. Some nurses tried their best to follow policy but also used their own discretion when deciding if a patient should be seen in-person: *“I would follow the public health guidelines… put out by (the regional health authority) as what we had to do policywise. But I still think I saw patients that maybe we were supposed to see virtually, and I would just put on (the PPE) I'm supposed to”* (BC13 NP). Likewise, in Nova Scotia, a nurse described taking the appropriate steps to protect themselves (e.g., PPE), but never turning away anyone who wanted to be seen in-person: *“… even if the patient was screening positive, when we were trying to do virtual, I never turned anybody away. So, if they wanted to come in-person, we just dressed as if they had COVID-19, in PPE, and we would see them”* (NS07 NP). In contrast, a nurse from Newfoundland and Labrador described how patients who had any risk of being exposed or infected were strictly seen virtually: *“… we had to go through a series of questions about the known symptoms of COVID-19 and possible exposures… If there was any risk whatsoever, the visit would not happen, it would have to be done over the phone or something”* (NL01 RN). It is important to note that these decisions may have also been influenced by the stage and epidemiology of the pandemic in each region. The triage process could also include consultations with physicians, as noted by a LPN in Nova Scotia:

*“We would triage and if they needed to be seen and they met the guidelines… you'd ask them all the questions if they had a cough, headache, sore throat, any of that, if they traveled or whatever. Then we would go to the physician and they would decide, “yes, they need to be seen” or “no, they need to go to the ER (emergency room)””* (NL09 LPN).

Notably, IPAC guidelines introduced constraints to clinic waiting rooms, as staff reduced the number of available seats to increase the physical distance between individuals. These changes limited the number of patients who could be seen in-person on any given day. In line with these considerations, a RN from British Columbia described how she kept the patient's best interest in mind when deciding whether or not they should be seen in-person, which could sometimes be met with hesitancy from clinic administrators:

*“…the clinic was hesitant about me bringing a bunch of (patients) in because of the limitations in the waiting room… But my bosses told me they didn't care. Like if I want to bring people in office, I bring people in office … I'm going to do what I'm going to do and that's whatever is best for my patients”* (BC05 RN).

### 4.2. Increased Workload

The implementation of IPAC policies increased nurses' workload due to (1) changes in workflow and (2) frequent updates in guidelines.

#### 4.2.1. Changes in Workflow

The implementation of IPAC protocols required nurses to complete new, time-intensive tasks that changed the normal rhythm of clinic operations and magnified the impact of previously routine issues such as missed or late appointments. In addition to providing primary care for their patients, many nurses participated in various IPAC activities including patient screening, cleaning, and the procurement and management of PPE supplies. For example, a nurse in Nova Scotia described their increased responsibilities due to their role in the procurement and management of IPAC–related supplies:

“*I also was responsible for our IPAC stuff. So, I had responsibility for the ordering of masks and gowns and gloves, and making sure that we had an area for them, and making sure that people knew how to use them, and that the front-end staff knew what to do with them… (I was also responsible for ordering) hand sanitizer and making sure we had the right cleaners to clean the rooms”* (NS08 RN).

These additional tasks were described as taking up a considerable amount of time: *“… screening, obviously that took a lot of time… obviously, the cleaning, the amount of, disinfecting (we had to do) in between patients… (we were) cleaning all the chairs and the blood pressure machines and your stethoscope every time…”* (ON12 RPN). These changes in workload were compounded by the reduced availability of staff to assist with the increasing workload in the clinic: *“… it was still very busy… making sure everything is completely wiped down between (visits)… the screening, that was a very hard one too because we had such minimal staff so we had to pull one person (to) cover each day”* (ON27 RPN). Nurses also described how IPAC protocols increased the number of tasks per patient visit, as each in-person appointment required multiple rounds of screening, donning and doffing of PPE, and thorough cleaning of supplies and surfaces. These additional responsibilities impacted the workload and the time required for each appointment:

*“… It was definitely more of an increase in workload. I mean, if you had five people come into the clinic in the afternoon that you were seeing face-to-face in the pandemic, it basically took you the same amount of time as if you were seeing 10 (prepandemic). By the time you put on PPE, put them in the proper room, wipe down the room, separated them, got them to complete screening, I mean, patients were probably in the clinic for an hour”* (NL06 NP).

The protocols also had the potential to reduce clinic capacity by limiting the number of patients that could be seen in-person on any given day. In Ontario, a RPN described how IPAC impacted their clinic's ability to effectively manage influenza vaccination clinics:

*“we'd run (flu shot) clinics… it was basically walk-in and we would (vaccinate) 300 (patients) in a three-hour window. And it was just like a well-oiled machine the way we did it for years and then when COVID-19 hit (and) we're like, well, we can't do that…. (because we) had people lined up in the hallways back-to-back. So, then we had to book off days where no physicians were in the office and it was booked appointments and six at a time. Everyone got screened outside, they came and spread out over the office, you gave them their shot, they waited in their chair, they left, you cleaned the chair, you brought in the next six. Like, so, it was just not efficient”* (ON12 RPN).

The increase in tasks per patient also made it more challenging for nurses when patients did not show up for appointments, as they had often exerted considerable effort to prepare and adhere to IPAC protocols: *“… we were all doing so many extra activities… the wiping down (of surfaces) … And just trying to procure supplies and do all those other things. So, when people were not showing up, it was having quite a negative effect (on morale)”* (NS08 NP). It is important to note that these changes could impact clinics and nurses in different ways. The initial development and need to align IPAC policies and protocols with the guidance from public health organizations were described by one nurse as being particularly taxing; however, she noted that the clinic became less busy later on due to the advent of virtual care:

*“… it was a lot of work initially getting all of those things set up for the clinic and making sure that the clinic was running in a way that met the standards and the guidelines that the medical health officer had set out. But slowly, as we moved more into that stage where we were asked to stay inside and not go out as much, the clinic also became less busy… So, a lot of that work that I was doing really kind of slowed down …”* (BC10 RN).

#### 4.2.2. Frequent Updates in Guidelines

The changes in workload were sometimes compounded by the need to align clinic policy with rapidly evolving guidelines from regional health authorities. This was frequently described by nurses in Nova Scotia, many of whom were employees of the Nova Scotia Health Authority. In these cases, the health authority provided them with public health updates that other clinic members did not have access to, highlighting how differing practice models sometimes resulted in unequal access to pandemic information: *“I'm Health Authority, (the others) are not… The office manager was relying on me to make sure that we knew what was being used and what was safe to be used, because they didn't have that information coming to them”* (NS08 RN). These nurses also became responsible for incorporating updates into their clinic's protocols:

*“There were some gray areas and the guidelines changed from time to time… There were many versions of the screening questions, so we had to make sure that we were offering the right questions. So, that became part of our morning routine, checking to see if there were any new emails with guidelines, which changed all the time”* (NS01 LPN).

The changing guidelines could create more work for nurses, sometimes leading to redundancy and inefficient use of time. In Ontario, NPs described their experience in developing screening protocols that turned out to not be reflective of changing public health guidelines, thereby highlighting a need for more proactive and timely guidance:

*“(We were asking) “have you traveled?” or “do you have symptoms?” I remember we would have a “yes, yes” pathway and a “yes, no” pathway and a “no, yes” and a “no, no” (pathway). We had four different pathways and we would literally come out of the three-hour meeting (solidifying) everything and the (public health) guidance had changed (during) the time that we were in the meeting. So, I'm sure I'm not unique in (experiencing) that”* (ON17 NP).

## 5. Discussion

Through qualitative interviews, we explored the roles nurses played in implementing and promoting IPAC in primary care settings during the COVID-19 pandemic. The nurses interviewed in our study were involved in developing and operationalizing IPAC policies and protocols for their clinic, teaching and educating different team members, and carrying out virtual-care decision-making. Implementing IPAC increased nurses' workload due to the upsurge of tasks associated with in-person patient visits and the repeated need to realign clinic policies with ever-changing guidelines from regional and public health authorities. Similar findings have been reported in studies from South Africa and the United Kingdom (UK), where rapidly evolving IPAC guidelines left nurses feeling poorly supported [34, 35]. In addition, our findings are consistent with a Canadian study that focused on the responsibilities of family physicians during the COVID-19 pandemic [6].

The development, implementation, updating, and ongoing monitoring of IPAC (including triaging for virtual care) were major responsibilities of primary care nurses during the pandemic. Although the application of IPAC procedures is listed as one of the entry-level competencies required for RNs to practice in a variety of settings [36], it is notably absent from descriptions of the routine activities of primary care nurses [37–40]. However, IPAC is described as part of the routine activities of public health nurses [41].

The ability of primary care nurses to develop and implement IPAC procedures highlights their capacity to collate, critique, synthesize, and apply evidence in creating and implementing programs. These roles are outlined in nursing competency documents using terms such as “professionalism,” “quality assurance,” “evaluation and research [42, 43],” “evidence-based practice [40],” or “continuous learning and research” [44]. These roles will likely remain prominent post-pandemic as there will be a need to maintain high standards of infection control to prevent future outbreaks [45], which may involve increased training, updated protocols, and continuous monitoring of infection rates. Furthermore, the integration of virtual care has been shown to be effective in reducing the risk of infection by minimizing in-person contact, a practice that is expected to persist [46, 47]. With the heightened awareness of IPAC and the ongoing use of virtual care, these roles and responsibilities are likely to become recognized as part of routine nursing practice in primary care, following the COVID-19 pandemic.

Our findings shed light on one of the sources behind increased workloads for healthcare providers during the pandemic [33, 35, 48]. In a recent survey of healthcare providers in Canada, 87.3% of nurses reported an increased workload during the pandemic [33], which in turn has been attributed as a cause of higher rates of burnout among nurses [49]. In Canada, approximately 92% of nurses reported more stress on the job during the pandemic [33]. Previous literature also details how pandemic-related factors and experiences impacted the mental well-being and morale of primary care nurses [37, 50–53]. Consistent with a study from the UK [35], we found that the need to develop, implement, and update IPAC protocols was time-consuming, especially in response to evolving directives from regional and public health authorities. Studies from Canada and elsewhere have noted that primary care providers were overwhelmed by the volume of conflicting information sent to them by various health, professional, and regulatory agencies [6, 11, 12, 54–63]. While information related to a novel disease (i.e., its epidemiology, clinical presentation, and management) may not be known in advance, information needed to adapt primary care settings and services, such as IPAC procedures and clinical safety, privacy, and triaging guidance for virtual care [64–67] can be anticipated and prepared in advance of the next pandemic. These findings underscore the need for better pandemic preparedness planning for primary care.

### 5.1. Study Limitations

The findings from this study should be interpreted in the context of its limitations. The data may have been impacted by social desirability bias, whereby participants feel pressure to present their experiences in a way that is deemed acceptable by societal standards [68]. We also limited recruitment and data collection to four health regions in Canada. The findings may, therefore, not reflect the experiences and IPAC roles of primary care nurses working in different regions or practices.

## 6. Conclusions

Nurses played an integral role in implementing IPAC across primary care settings in Canada during the COVID-19 pandemic. The upstream touchpoint of nurses, prior to physician assessment, places nursing IPAC readiness and leadership at the forefront of primary care pandemic preparedness. The nurses we interviewed described operationalizing public health guidance to reflect unique clinic contexts. They allowed other clinic staff to implement and follow IPAC policies and protocols through teaching and education and by using clinical expertise to make important decisions regarding which patients should be seen in-person vs. virtually. These experiences underscore primary care nurses' abilities to use their knowledge and training to effectively implement successful IPAC procedures.

This study's findings also highlight the increased workload stemming from poor pandemic preparedness in primary care. Nurses stepped in to fill gaps in available guidelines to safeguard primary care settings from the risk of COVID-19 transmission. The findings highlight the need for better pandemic planning to support primary care. IPAC guidance documents, suitable for primary care settings and informed by the experiences from the COVID-19 pandemic, should be included in future pandemic preparedness plans.

## Figures and Tables

**Table 1:** Demographic characteristics of study participants.

Demographic characteristics	British Columbia *N* = 13	Ontario *N* = 27	Nova Scotia *N* = 20	Newfoundland and Labrador *N* = 16	Total *N* = 76
Gender^∗^, *n* (%)					
Men or nonbinary	2 (15.4)	1 (3.7)	1 (5.0)	0 (0)	4 (5.3)
Women	11 (84.6)	26 (96.3)	19 (95.0)	16 (100)	72 (94.7)
Nurse type, *n* (%)					
RPN/LPN	0 (0)	9 (33.3)	1 (5.0)	5 (31.3)	15 (19.7)
RN	11 (84.6)	9 (33.3)	11 (55.0)	6 (37.5)	37 (48.7)
NP	2 (15.4)	9 (33.3)	8 (40.0)	5 (31.3)	24 (31.6)
Community size^∗∗^, *n* (%)					
Rural	1 (7.7)	10 (37.0)	11 (55.0)	6 (37.5)	28 (36.8)
Small urban	3 (23.1)	5 (18.5)	6 (30.0)	0 (0)	14 (18.4)
Urban	9 (69.2)	12 (44.4)	3 (15.0)	9 (56.3)	33 (43.4)
Mixed	0 (0)	0 (0)	0 (0)	1 (6.3)	1 (1.3)
Years in practice, mean (SD)	17.7 (11.2)	13.6 (10.1)	15.6 (11.3)	14.0 (9.8)	14.9 (10.3)

^∗^Gender was asked as an open-ended question. Nonbinary participants were amalgamated to maintain participant confidentiality and grouped with men to represent minority genders in our participant sample/the nursing profession.

^∗∗^Rural < 10,000 population; small urban = 10,000–99,999 population; urban > 100,0000 population [33]; mixed: participants reported practicing in more than one community that involved rural and urban populations.

**Table 2:** Summary of major themes and subthemes.

Theme 1: Nurse-led transformation of clinic operations • Policy and protocol development • Teaching and education • Triaging virtual and in-person care
Theme 2: Impact on workload • Changes in workflow • Frequent updates in guidelines

## Data Availability

The datasets analyzed for this study are not publicly available due to the need to maintain participant confidentiality; however, a portion of these data may be available from the corresponding author upon reasonable request.

## Supporting Information

Supporting File 1. Interview Guide.
